# Syncope due to vitamin-B12 deficiency – case report

**DOI:** 10.25122/jml-2024-0305

**Published:** 2025-05

**Authors:** Josef Finsterer

**Affiliations:** 1 Neurology and Neurophysiology Center, Vienna, Austria

**Keywords:** vitamin-B12 deficiency, syncope, sick sinus syndrome, autonomic neuropathy

## Abstract

To our knowledge, a patient with recurrent syncope due to vitamin B12 deficiency without hematologic manifestations has not been reported before. We present the case of an 83-year-old man who experienced a third episode of syncope, characterized by sudden loss of consciousness without convulsions and rapid recovery. His medical history included ischemic stroke 22 years earlier and multiple bowel resections following cholecystectomy 21 years ago. The examination revealed a severe vitamin B12 deficiency and bradycardia on ECG. The examination for heart disease was inconclusive. The syncope was attributed to a vitamin B12 deficiency and consecutive cardiac autonomic neuropathy (CAN) after other causes for the syncope were sufficiently ruled out. Syncopes did not recur after adequate vitamin B12 supplementation. This case illustrates that partial bowel resection may lead to severe vitamin B12 deficiency, which can result in CAN. Disturbed autonomic innervation of the cardiac conduction system can lead to bradycardia, which, in turn, can result in syncope. Patients with a history of complete or partial bowel resection should undergo regular monitoring of vitamin B12 levels to prevent potentially symptomatic deficiency.

## INTRODUCTION

Vitamin B12 deficiency may result from insufficient dietary intake, impaired bioavailability, or malabsorption and typically presents with hematologic and/or neurologic abnormalities [1]. Neurological manifestations of vitamin B12 deficiency include developmental delay, dizziness, seizures, hypotonia, tremor, ataxia, tingling, paresthesias, visual disturbances, fatigue, difficulty concentrating, and syncope [2]. These symptoms generally resolve within weeks following appropriate vitamin B12 supplementation [2]. Syncope as a complication of vitamin B12 deficiency has been reported only rarely [3,4]. Syncope in vitamin B12 deficiency may be due to autonomic neuropathy or severe megaloblastic anemia [1]. To date, recurrent syncope from B12 deficiency-related cardiac autonomic neuropathy (CAN) without hematologic findings has not been reported.

## CASE REPORT

An 83-year-old man (161 cm, 77 kg) was admitted after experiencing a third syncopal episode. The event occurred without physical exertion or prodromal symptoms. While standing in his kitchen, he suddenly collapsed backward and lost consciousness for approximately one minute. There was no loss of bladder or bowel control, tongue or cheek biting, or convulsions. He regained consciousness quickly and oriented himself without confusion. He reported no palpitations, diaphoresis, or nausea, and the ambient temperature in the kitchen was normal. Previous syncopal episodes had occurred in 2023 and February 2024.

In 2003, he underwent a cholecystectomy complicated by iatrogenic bowel injury, necessitating multiple bowel resections and mesh placement for recurrent hernias between 2003 and 2005. These procedures resulted in short bowel syndrome and chronic diarrhea, which resolved two years prior to presentation. He also reported cataract surgery on both sides, hypoacusis requiring a hearing aid, hyperlipidemia, a torn ligament in his left ankle, paraproteinemia, and an ischemic stroke with left-sided weakness in 2002, which has since resulted in impaired fine motor function. The medical history revealed no evidence of peripheral or coronary artery disease. He did not follow a specific diet and had an omnivorous diet. His home medication only included acetylsalicylic acid.

Clinical neurological examination revealed unrounded pupils, bilateral hypoacusis, dry tongue, and small palpebral fissures. Discrete proximal weakness, diminished biceps tendon reflexes, diminished position sense, and a passing finger-nose test (right>left) were noted in the upper limbs. The paravertebral muscles were tender to palpation. The lower limbs showed reduced foot extension (M5-) on the left side, a positive Lasegue sign on the left side at 80 degrees, reduced tendon reflexes, mild ataxia on both sides, hypoesthesia, hypoalgesia and pallhypesthesia on both sides. Both Romberg and tandem gait tests revealed a marked, non-directional tendency to fall.

On admission, blood pressure was 130/87 mmHg. Electrocardiography showed sinus bradycardia and ventricular ectopy. Continuous telemetry recorded frequent ventricular ectopic beats, couplets, and triplets but no atrial fibrillation, with a minimum heart rate of 34 bpm. Carotid ultrasound and transthoracic echocardiography were normal. Coronary angiography was unremarkable. The oto-rhino-laryngologic examination was non-revealing. The examination of the suspected polyneuropathy revealed a reduced compound muscle action potential (CMAP) in the median and peroneal nerves, as well as a reduced sensory nerve action potential (SNAP) and a slowing in the sural nerves. Cerebral CT showed an old partial infarction in the area of the right middle cerebral artery and bilateral calcifications of the basal ganglia but no acute pathology. Cerebral MRI showed a post-ischemic lesion on the right and a frontal arachnoid cyst on the left. No epileptiform discharges were detected on EEG. X-ray of the spine revealed marked spondylosis deformans of the thoracic spine, anterolisthesis L4/5, and osteochondrosis L5/S1. Blood examination revealed normal red and white blood cell count, normal hemoglobin and mean corpuscular volume, mild iron deficiency, and low transferrin saturation. proBNP was slightly elevated, but troponin was normal. Surprisingly, a marked vitamin B12 deficiency of <50 pg/ml (normal: 180-940 pg/ml) was found. Homocysteine was elevated to 9.8 µmol/L (normal: <8µmol/L). After intravenous vitamin B12 substitution, the low serum level normalized within 10 days, and the symptoms disappeared within two weeks. The course during hospitalization was uneventful, and syncopes did not recur.

## DISCUSSION

The patient is of interest because of recurrent syncope due to vitamin B12 deficiency, which impaired the autonomic innervation of the heart. In addition to syncope, autonomic dysfunction due to autonomic neuropathy may manifest as brain fog, dizziness, photosensitivity, dysphagia, arterial hypotension, tachycardia, bowel incontinence or constipation, urinary incontinence or retention, impotence, anhidrosis/hyperhidrosis, or exercise intolerance [5]. The patient did not exhibit these other manifestations of autonomic neuropathy. The syncope was attributed to vitamin B12 deficiency, as other causes of syncope could be ruled out. There was no evidence of acute stroke, cerebral hemorrhage, seizure, carotid stenosis, or cardiac disease. Prolonged ECG monitoring and inpatient telemetry revealed sinus rhythm with bradycardia and ventricular ectopy but no significant conduction abnormalities or arrhythmias. Transthoracic echocardiography was unremarkable, and coronary angiography excluded obstructive coronary artery disease.

The polyneuropathy was attributed to a vitamin B12 deficiency, as other causes could be ruled out. There was no evidence of diabetes, alcohol abuse, renal insufficiency, immunological diseases, Guillain-Barre syndrome, poisoning, or malignant diseases. Vitamin B12 deficiency is a well-recognized cause of peripheral neuropathy [6], and there is growing evidence of its role in autonomic dysfunction. There is also evidence that metformin-induced vitamin B12 deficiency can cause or exacerbate autonomic neuropathy or CAN [7]. In a study of 469 patients with diabetes who were screened for CAN, vitamin B12 levels were inversely related to the presence of CAN [8]. The family history of the index patient was negative for hereditary neuropathy. The vitamin B12 deficiency in the index patient was attributed to his resection of the large and small intestines. It is known that resection of the stomach or small intestine can be complicated by vitamin B12 deficiency [9]. Similar complications have been reported in patients undergoing bariatric surgery [10].

## CONCLUSION

In conclusion, this case highlights that partial bowel resection may result in severe vitamin B12 deficiency and secondary CAN, potentially leading to bradycardia and syncope. In patients who have undergone complete or incomplete bowel resection, close monitoring of vitamin B12 levels is necessary to prevent overlooking vitamin B12 deficiency, which typically presents with a mild course if diagnosed and treated promptly.

## Data Availability

Data that support the findings of the study are available from the corresponding author.
